# Genomic profile of metastatic breast cancer patient-derived xenografts established using percutaneous biopsy

**DOI:** 10.1186/s12967-020-02607-2

**Published:** 2021-01-06

**Authors:** Seongyeong Kim, Dongjin Shin, Ahrum Min, Minjung Kim, Deukchae Na, Han-Byeol Lee, Han Suk Ryu, Yaewon Yang, Go-Un Woo, Kyung-Hun Lee, Dae-Won Lee, Tae-Yong Kim, Charles Lee, Seock-Ah Im, Jong-Il Kim

**Affiliations:** 1 Cancer Research Institute, Seoul National University, Seoul, Korea; 2 Department of Biomedical Sciences, Seoul National University College of Medicine, Seoul, Korea; 3 Biomedical Research Institute, Seoul National University Hospital, Seoul, Korea; 4 Medical Research Center, Genomic Medicine Institute (GMI), Seoul National University, 101 Daehak-ro, Jongno-gu, Seoul, 03080 Korea; 5 Ewha Institute of Convergence Medicine, Ewha Womans University Mokdong Hospital, Seoul, Korea; 6 Department of General Surgery, Seoul National University Hospital, Seoul, Korea; 7 Department of Pathology, Seoul National University Hospital, Seoul, Korea; 8 Translational Medicine, Seoul National University College of Medicine, Seoul, Korea; 9 Department of Internal Medicine, Chungbuk University Hospital, Cheong-Ju, Korea; 10 Department of Internal Medicine, Seoul National University Hospital, Seoul National University College of Medicine, 101 Daehak-ro, Jongno-gu, Seoul, 03080 Korea; 11 Department of Life Science, Ewha Womans University, Seoul, Korea; 12 The Jackson Laboratory for Genomic Medicine, Farmington, Connecticut USA; 13 Precision Medicine Center, The First Affiliated Hospital of Xi’an Jiaotong University, Xi’an, China

**Keywords:** Metastatic breast cancer, Patient-derived xenograft, Whole-exome sequencing

## Abstract

**Background:**

Metastatic breast cancer (mBC) is a complex and life-threatening disease and although it is difficult to cure, patients can benefit from sequential anticancer treatment, including endocrine therapy, targeted therapy and cytotoxic chemotherapy. The patient-derived xenograft (PDX) model is suggested as a practical tool to predict the clinical outcome of this disease as well as to screen novel drugs. This study aimed to establish PDX models in Korean patients and analyze their genomic profiles and utility for translational research.

**Methods:**

Percutaneous core needle biopsy or punch biopsy samples were used for xenotransplantation. Whole exome sequencing and transcriptome analysis were performed to assess the genomic and RNA expression profiles, respectively. Copy number variation and mutational burden were analyzed and compared with other metastatic breast cancer genomic results. Mutational signatures were also analyzed. The antitumor effect of an ATR inhibitor was tested in the relevant PDX model.

**Results:**

Of the 151 cases studied, 40 (26%) PDX models were established. Notably, the take rate of all subtypes, including the hormone receptor-positive (HR +) subtype, exceeded 20%. The PDX model had genomic fidelity and copy number variation that represented the pattern of its donor sample. *TP53, PIK3CA, ESR1,* and *GATA3* mutations were frequently found in our samples, with *TP53* being the most frequently mutated, and the somatic mutations in these genes strengthened their frequency in the PDX model. The *ESR1* mutation, *CCND1* amplification, and the APOBEC signature were significant features in our HR + HER2- PDX model. Fulvestrant in combination with palbociclib showed a partial response to the relevant patient’s tumor harboring the *ESR1* mutation, and *CCND1* amplification was found in the PDX model. AZD6738, an ATR inhibitor, delayed tumor growth in a relevant PDX model.

**Conclusions:**

Our PDX model was established using core needle biopsy samples from primary and metastatic tissues. Genomic profiles of the samples reflected their original tissue characteristics and could be used for the interpretation of clinical outcomes.

## Background

Breast cancer is the most prevalent cancer in women, and metastatic breast cancer (mBC) remains a difficult-to-treat disease, with a 5-year survival rate still below 40% [1]. Treatment decisions are largely based on well-classified molecular markers based on immunohistochemistry (IHC) for hormone receptors (HRs) and IHC with fluorescence in situ hybridization (FISH) for human epidermal growth factor receptor 2 (HER2). However, clinical molecular subtyping is insufficient when patients fail to respond to standard case therapies, and precise molecular markers are needed to select targeted drugs for each patient and to implement precision medicine [2]. Moreover, tumor heterogeneity results in differences even in the same subtype [3]. Due to insufficient molecular markers and drug resistance, various chemotherapeutic drug combinations used for the treatment of mBC have been unsuccessful [4]. Thus, there is an eager need to identify novel molecular markers using better techniques and understand the characteristics of the individual tumor.

Patient-derived xenograft (PDX) models are known to provide a more accurate reflection of tumor biology than cell lines [5]. Previous studies have reported that established PDX models retain the histological and genetic characteristics of their donor tumor; therefore, it is assumed that this model has the potential to predict clinical outcomes and can be used to screen newly developed drugs [6]. Moreover, the PDX model is also utilized for precision medicine and therapeutic marker discovery. The key technology for precision medicine is high-resolution sequencing, and the genome data obtained from it can provide insight to understand each tumor and to select precise treatments. It is not easy to obtain a sufficient amount of tumor tissue for multiple analyses from metastatic breast cancer; thus, the establishment of the PDX model can be a practical solution. Despite these advantages, the PDX model also has a few limitations. Primary surgical tissues are usually used to engraft the PDX model; hence, PDX models using metastatic tissues are scarce. The difference in take rates between the subtypes is also a critical issue. The take rate exceeds 50% in triple-negative breast cancer, which is an aggressive subtype; in contrast, the take rate in the estrogen (ER)- or progesterone receptor (PR)-positive subtype, which is relatively indolent, is usually less than 10% [6, 7]. In addition, there are still disparities in ethnic and racial distributions. In previous studies, most PDX models were established from Caucasian women, followed by Afro-American and Hispanic women [8]. PDX models from Asian breast cancers are relatively scarce, and their characteristics are underrepresented.

In this study, we report 40 PDX models established using percutaneous core needle biopsy or punch biopsy samples of Korean patients with mBC. The overall take rate was 27%. We analyzed the histological and genomic profiles of the patient tumor and engrafted PDX tissues to verify the similarity between the PDX model and donor tissue. After PDX establishment, the tumor tissues were analyzed using whole-exome sequencing (WES) to assess the mutation and copy number variation (CNV) patterns. Moreover, to evaluate the potential use of PDX in translational research, we tried to apply the information from the PDX models to the selection of drugs for some patients, and we investigated an inhibitor of ataxia telangiectasia and Rad3 related (ATR) using relevant models.

## Methods

### Patient recruitment and tissue collection

Patients diagnosed with locally advanced or metastatic breast cancer were enrolled in this study for PDX establishment and genomic profiling from 2014 to 2017. The patient tissues of the primary breast or various metastatic sites (Additional file 1: Table S1) were acquired by percutaneous needle biopsy and placed in RPMI 1640 tissue culture medium (Thermo Fisher Scientific Inc., Waltham, MA, USA) supplemented with 10 U/ml penicillin and 10 µg/ml streptomycin. The maximum portion of the tissue was used for mouse transplantation, whereas the remaining portion was used for genetic analysis. The study protocol was approved by the Institutional Review Board of Seoul National University Hospital (SNUH) (IRB No.: 1402-054-555).

### Establishment of the PDX mouse model

The xenograft experiment was performed after approval from the Institutional Animal Care and Use Committee. NOD.Cg-*Prkdc*^*scid*^* Il2rg*^*tm1Wjl*^/SzJ (NSG) mice were purchased from Jackson Laboratories (Bar Harbor, ME, USA). Fresh biopsy tissue was prepared within 30 min and was implanted into the mammary fat pad near the left leg of NSG mice within 1 h. The mouse was monitored until the tumor reached a size of 200 mm^3^ or for 6 months. When the xenograft tumor size exceeded 200 mm^3^, tumor xenografts were excised, and the tissue fragments were transplanted into other mice to increase the number of tumor-bearing mice or were preserved in liquid nitrogen for future engraftment. A portion of the tumor tissue was fixed in formalin for pathological analysis, and the remaining portion was preserved for genomic analysis.

Each sample was named as follows: 'IMT_' or 'T_' stands for patient tumor sample, and 'IMX_' or 'X_' stands for PDX tumor sample.

### DNA extraction, library preparation, and genome analysis

Genomic DNA was extracted from frozen tissue. Genomic DNA from PDX and patient biopsy tissues was prepared with a DNeasy Blood and Tissue Kit (Qiagen, Hilden, German). Genomic DNA from patient blood samples was prepared with a Qiagen Gentra Puregene Blood Kit. Agilent SureSelectXT Human All Exon V5 (Agilent Technology Inc., CA, USA) was used for DNA library preparation. All experiments were performed according to the manufacturer’s instructions.

**Fig. 1:**
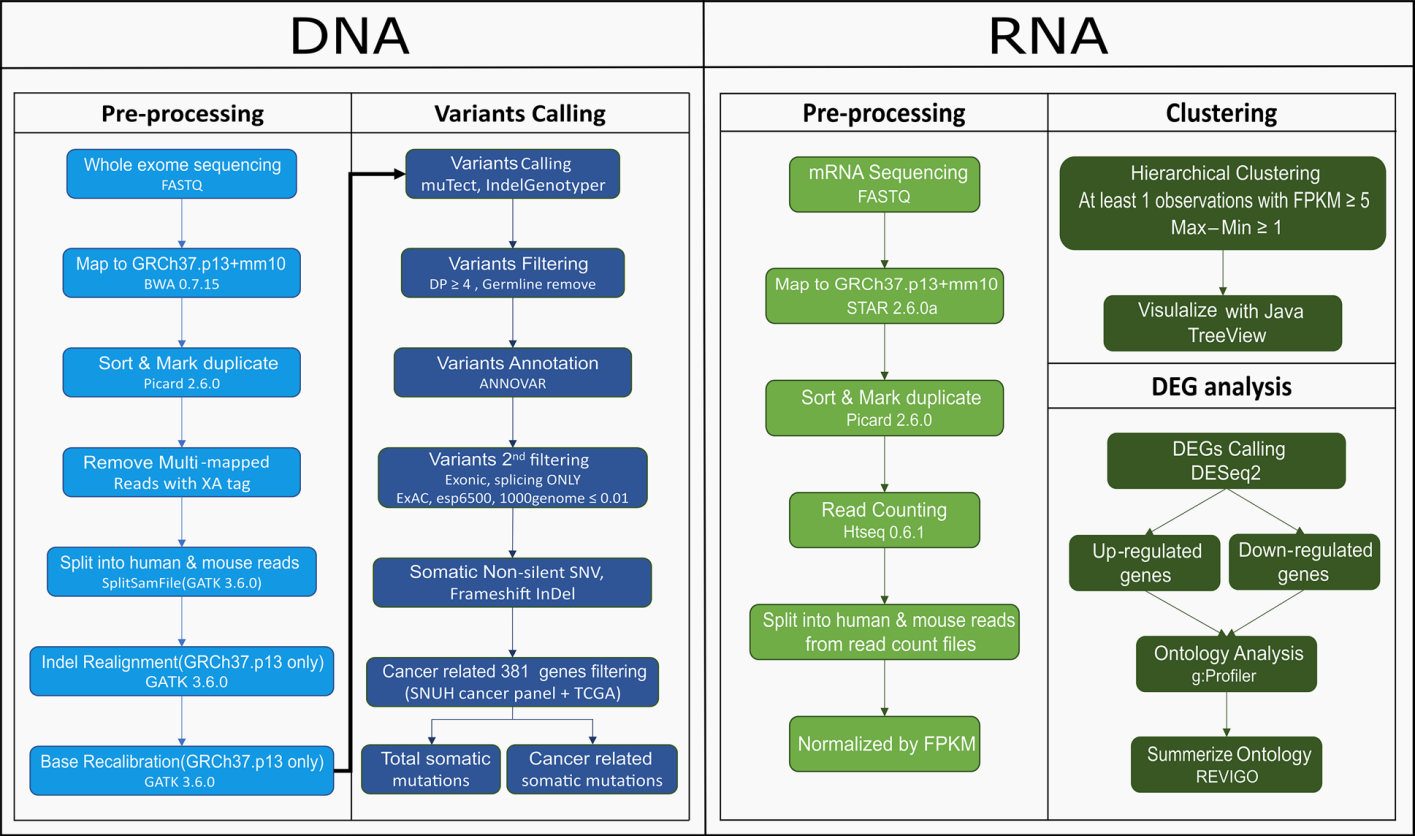
Flow chart of WES and transcriptome data processing. Detailed information on the data mapping process and the variant calling process is given

WES was performed using the HiSeq 2500 system (Illumina). WES reads were mapped to the combined reference for human GRCh19 and mouse mm10 genome versions using BWA [9]. We used a combined reference, as mixed samples from different species could be obtained. Multiple mapped reads were discarded because they likely span the human and mouse references. Then, we separated BAM files using the human reference to obtain human specific reads. Additional preprocessing followed the recommendations of the Genome Analysis Tool Kit (GATK) [10]. The genomic data analysis process is depicted as a flow chart in Fig. 1.

The mean depth of our sequencing data was 150X in the tumor tissue of the patient and the PDX and 100X in the blood sample. The samples achieved an average of 97.87% of the targeted exome bases covered to a depth of 10X or greater. The detailed information on sequencing data from individual samples is attached in Additional file 2.

### Variant calling and copy number alteration analysis

Single nucleotide polymorphisms (SNPs) and small insertions and deletions (Indels) were analyzed using MuTect (1.1.7) and IndelGenotyper provided by GATK (3.6.0), respectively [11]. Variants with at least four read depths were selected, and the nonsilent somatic mutation was assessed using an in-house filtering method (total depth ≥ 4, exonic splicing, or nonsynonymous SNV or frameshift InDel or population DB frequencies ≤ 0.01). Additionally, various population study databases (ExAC (Exome Aggregation Consortium) [12], esp6500 (Exome Sequencing Project v. 6500) [13], and the 1000 Genomes project [14]) were used to filter common variants. We distinguished germline mutations by paired sequencing of blood samples. Moreover, we investigated whether the CNVs were maintained in the PDX models. CONIFER [15] was used to enumerate the read fragments in the WES data. The logarithm of blood and tumor sample sequencing data was used for CNV analysis, and the somatic CNVs were segmented with “DNA copy.” B allele frequencies (BAFs) were selected by MuTect and visualized with Integrative Genomics Viewer (IGV).

### Mutational signature analysis

The mutational signature was analyzed using deconstructSigs [16], which allows an individual analysis of each sample. The mutation fractions were calculated from the somatic mutations from each sample. Extracted signatures were characterized based on 96 trinucleotide contexts. To efficiently distinguish the signature, cosine similarity was obtained by comparison with the COSMIC signatures [17].

### RNA sequencing and normalization

The tumor tissues obtained from the PDX were used for transcriptome sequencing. TruSeq v2 (TruSeq RNA Library Prep Kit v2, Illumina Inc., CA, USA) was used to prepare the RNA library. Transcriptome sequencing data were mapped to the same reference as WES data using STAR aligner [18]. Subsequent processing was performed as the Best Practices workflow for RNA-seq using GATK. Gene expression levels were quantified for BAM files by fragments per kilobase of exon per million mapped reads (FPKM) using HTSeq-count [19]. The transcriptome data analysis process is depicted as a flow chart in Fig. 1, and the detailed information of transcriptome data from individual samples is attached in Additional file 2.

The transcriptome data were used for subtyping the PDX samples into 4 subtypes (luminal A, luminal B, HER2-enriched, and basal like) according to previous methods [20].

### Differentially expressed genes and ontology analysis

The DESeq2 algorithm was used to determine the expression level change between *ESR1*-mutant and *ESR1* wild-type HR + samples [21]. g:Profiler, which is a web-based tool, was used to evaluate the ontology to identify the different pathways and their mechanisms [22], and REVIGO was used to reduce ontologies [23].

### Antitumor efficacy study of targeted agents using PDXs

AstraZeneca provided AZD6738, an ATR inhibitor [24]. This drug was administered by oral gavage once daily at a concentration of 50 mg/kg for 4 weeks. The tumor was measured every other day using calipers, and the volume was calculated with the following formula: [(width)^2^ × (height)]/2. Following 4 weeks of drug treatment, the mice were monitored to assess tumor growth.

## Results

### Patient characteristics

In total, 151 core needle biopsy tissues were obtained from 130 patients between 2014 and 2017 for the establishment of PDXs. The enrolled patients presented with locally advanced breast cancer or mBC during tissue collection. Of the 151 tissues, 38 were collected from the primary breast tumor site, and 113 originated from various metastatic tissues. The tumor subtype based on estrogen receptor (ER), progesterone receptor (PR), and HER2 expression was determined by IHC (aided by FISH for HER2). The HR + HER2- subtype was the most commonly observed (64/151, 42.4%), whereas the prevalence of the HR + HER2 + subtype was the lowest (18/151, 11.9%). Most of the tumor histologic types were infiltrating ductal carcinoma (IDC, 140/151, 92.7%), 7 (4.6%) were invasive lobular carcinoma (ILC), and 3 (2.0%) represented a mixture of IDC and ILC (Table 1). The characteristics of each individual and the corresponding PDX model are presented (Additional file 1: Table S2).

**Table 1:** Characteristics of the PDX specimen

Characteristic	Number of samples N = 151^a^(%)
Age (years)	
Median (range)	53 (28–78)
Biopsy site of origin	
Primary site (Breast)	38 (25%)
Metastatic site	113 (75%)
Tumor subtype	
HR + HER2-	64 (42.4%)
HR + HER2 +	18 (11.9%)
HR − HER2 +	28 (18.5%)
TNBC	41 (27.2%)
Histologic subtype	
Invasive ductal carcinoma	140 (92.7%)
Invasive lobular carcinoma	7 (4.6%)
Mixed ductal and lobular carcinoma	3 (2.0%)
Metaplastic carcinoma with matrix-producing and squamous cell carcinoma	1 (1.3%)

^a^The number of enrolled patients was 130

### The established PDX model was concordant with the tissue of origin

**Fig. 2:**
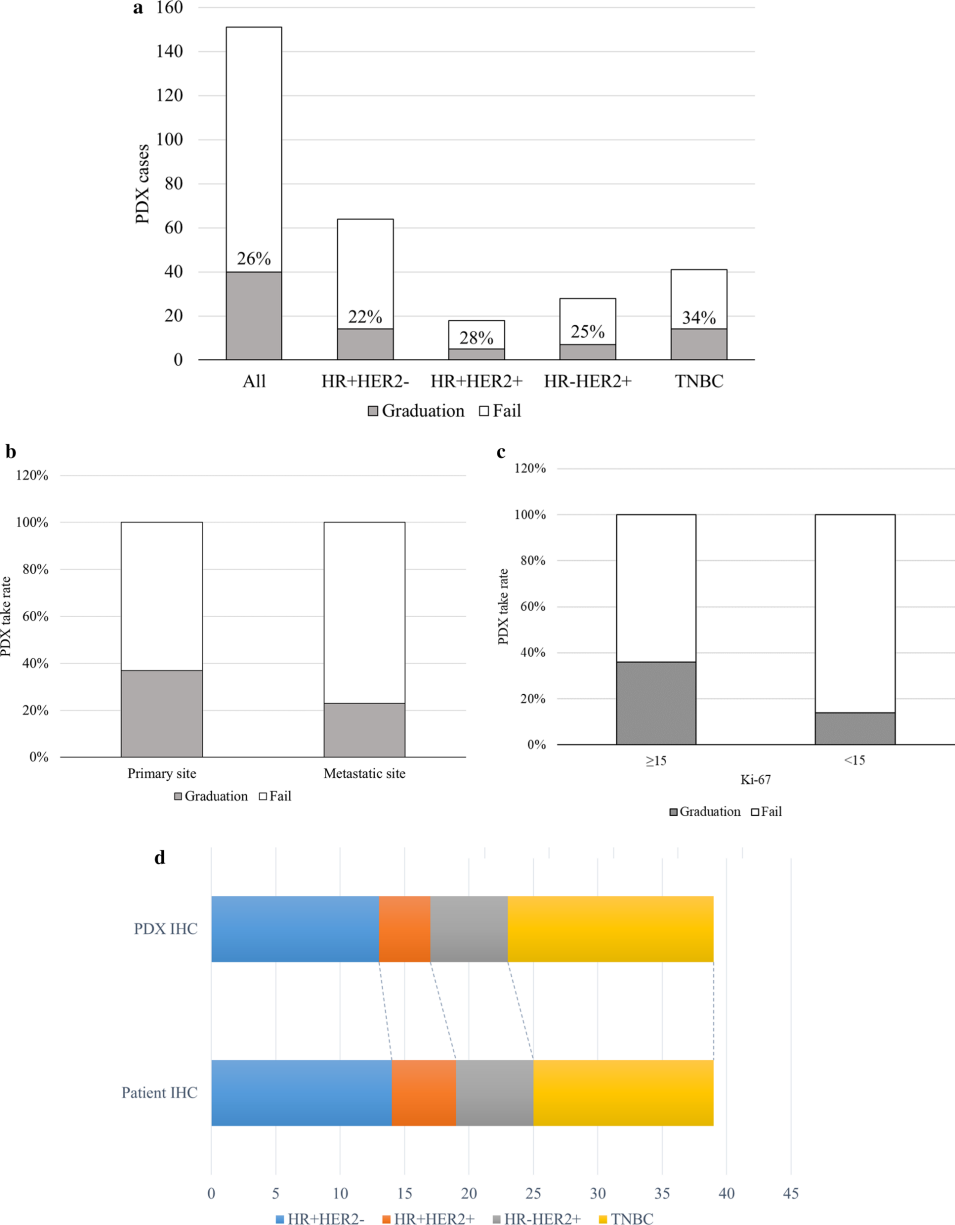

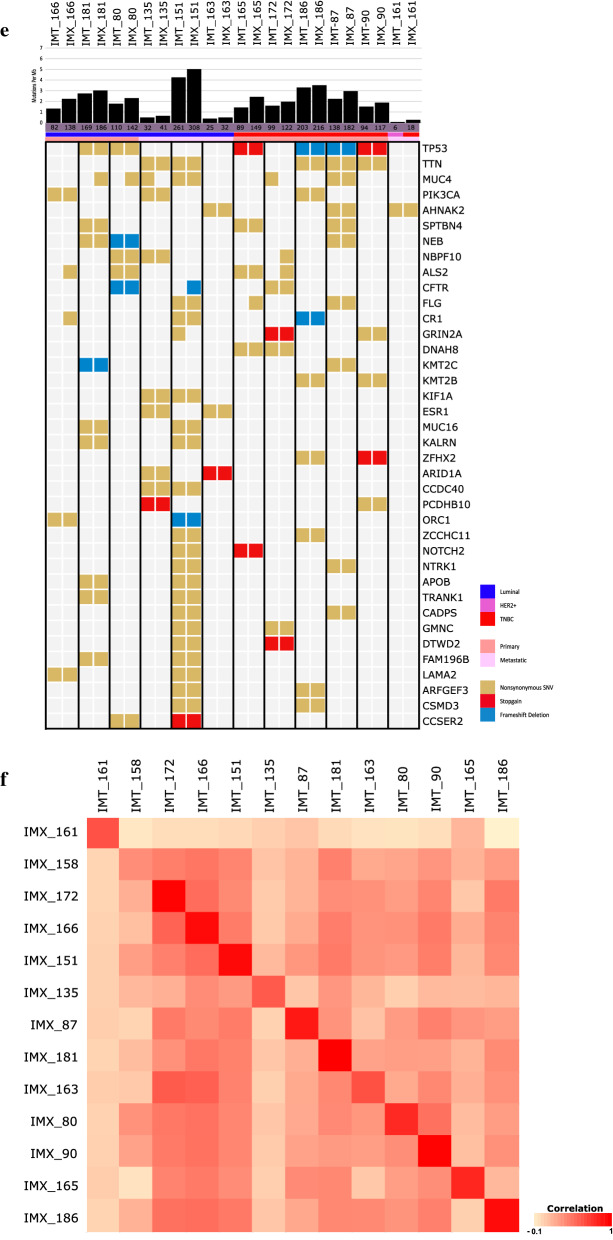
The statistics of PDX model establishment and fidelity between the PDX model and donor. **a** The total numbers of enrollment and take cases and those subdivided (enrollment cases and take cases) are represented by a bar graph. The take rate is also marked. **b** The take rate by tissue origin is represented by a bar graph. **c** The take rate by Ki-67 expression in patient tissues is represented by a bar graph. **d** The subtype composition of the established PDX model and its tissue of origin. The subtype was compared with PDX IHC analysis and clinical records. **e** The somatic mutation patterns of the patient tissue and its corresponding PDX model. The case number with IMT indicates the results of patient tissue analysis, and the case number with IMX indicates the results of PDX tissue analysis. The list of genes is ordered by mutational frequency, and the number of somatic mutations of each sample is marked in the figure. **f** CNV concordance between patient and PDX tissues. The correlation was determined by Pearson’s correlation analysis using the log2-fold change value of each sample

The PDX take rate in our system was 26% (40/151). We subdivided this rate according to the tissue subtype. The TNBC subtype exhibited the highest take rate (34%). Notably, the take rate of all subtypes exceeded 20% (Fig. 2a). The take rate of the PDX model using primary tissues tended to be higher than that using metastatic tissues (37% (14/38) vs. 23% (26/113), *p* = 0.114), although the difference was not statistically significant. The absolute case numbers were higher in the metastatic model (Fig. 2b). In particular, the take rate of the TNBC subtype using primary tumors was 60% (9/15), but that using metastatic site tumors was 19% (5/26). We further analyzed whether the degree of Ki-67 expression was relevant to the PDX take rate. Among the 151 cases, 85 were available for analysis. We divided these cases into two groups based on the median degree of Ki-67 expression (n = 15). The take rate was more than twice in the group of 15 and above compared to the other group (Fig. 2c). The histological characteristics were approximately 90% concordant between the primary and PDX tumors (Fig. 2d, Additional file 1: Fig. S1a).

The somatic mutation pattern was inspected to confirm the similarity between the PDX model and patient tissue (Fig. 2e). This result demonstrated that the PDX and patient genomes presented considerably identical mutations. We further analyzed whether the CNVs were altered in the PDX model. Using Pearson’s correlation analysis, we compared the CNVs between patient tumors and PDX tumors. As presented in Fig. 1f, the overall CNV patterns were well correlated between the tissue of origin and the PDX model (Additional file 1: Fig. S1c). These results indicate that our metastatic breast PDX model has high concordance with its origin.

### Genomic alterations in PDX samples

**Fig. 3:**
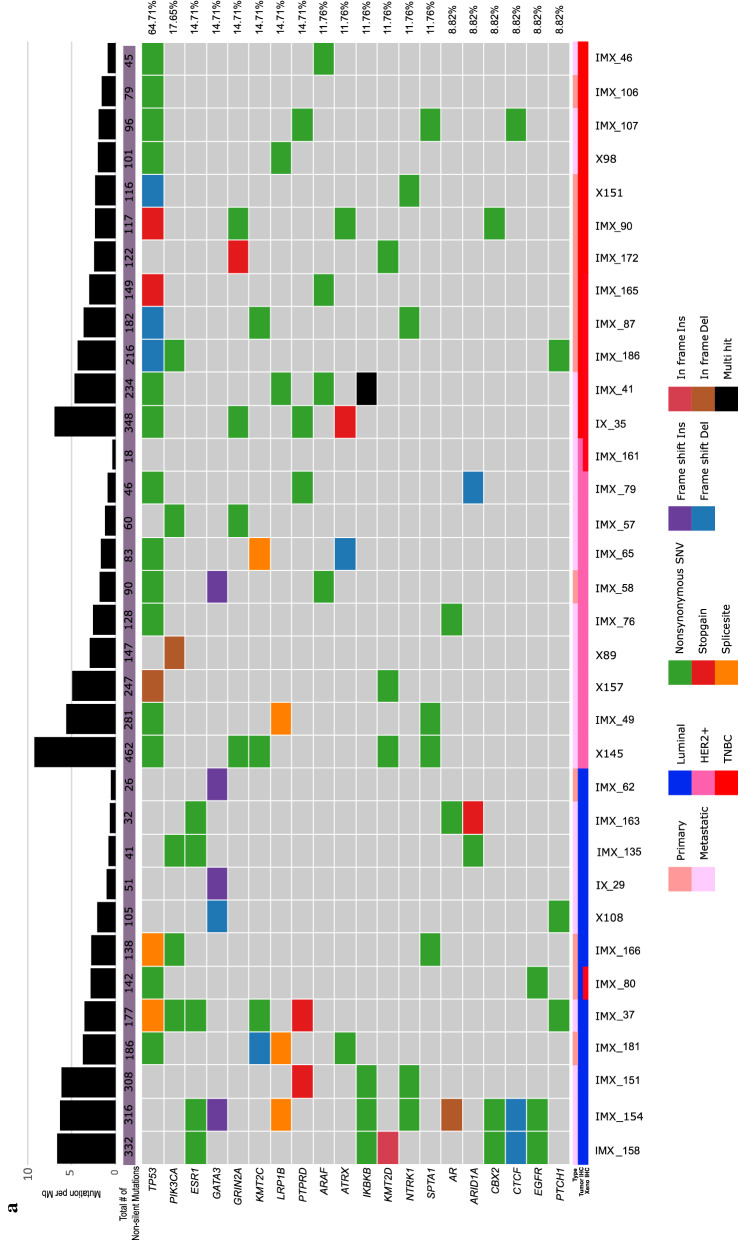

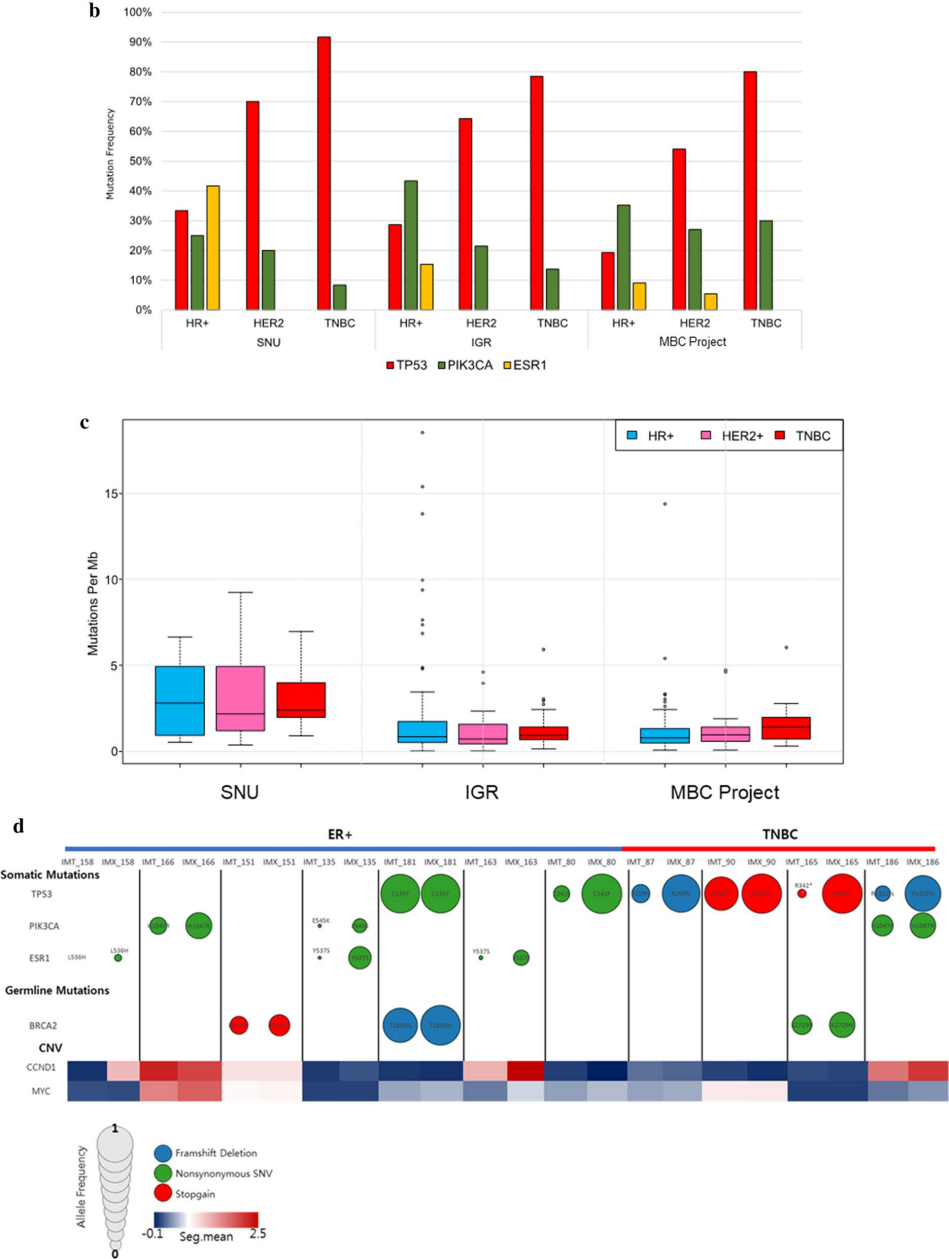
Genomic profile characteristics of the Korean metastatic breast PDX model. **a** Somatic mutations in our PDX model are arranged according to frequency. The samples are arranged by their subtype. The mutation burden of each sample is also presented. The genes are listed according to their mutation frequency, from high to low. **b** The mutation frequencies of *TP53*, *PIK3CA*, and *ESR1* were analyzed by subtype and are represented by a bar graph (a comparison with the other datasets is also shown). **c** Mutation burden of each subtype. **d** The mutation frequency of actionable mutation genes. The mutations that were detected in the patient and PDX tumor samples derived from the same patient were compared

The genomic variations in the PDX samples were analyzed using WES. We found that 6094 nonsilent mutations led to insertions and deletions. The range of nonsilent mutation counts in individual samples varied from 24 to 631. Next, we arranged the mutated gene list from high to low frequencies (Fig. 3a). *TP53* was the most commonly mutated gene among our samples, with a mutation rate of 64.7%. Unlike *TP53*, the mutation rate of other genes did not exceed 20%. The frequency of *PIK3CA* mutations was 17.6%, *ESR1* mutations were detected in 15% of all samples, and *GATA3* mutations were detected in 15% of all samples.

To evaluate the features of our models, the public datasets were analyzed and compared with our data. Specifically, 145 cases were from “the metastatic breast cancer project” [25], and 216 cases were from “the SAFIR01, SAFIR02, SHIVA, or Molecular Screening for Cancer Treatment Optimization (MOSCATO) prospective trials” [26]. The mutation frequencies of *TP53, KMT2C*, and *ESR1* were higher in our model, but the mutation frequency of *PIK3CA* was relatively low (Additional file 1: Fig. S2a). To eliminate the sample number differences of each subtype in the individual dataset, the mutation frequencies of three representative genes from each dataset were compared according to the subtypes. As presented in Fig. 3b, the frequency of *TP53* and *ESR1* mutations were significantly higher in our dataset compared with other datasets, whereas the frequency of *PIK3CA* mutations was lower. The mutation burden of our model was significantly higher than that of the other datasets (Fig. 3c). A comparison of the allele frequencies between the patient and PDX tumors revealed that the germline and somatic alterations in breast cancer-related genes were stably conserved and amplified in the PDX tumors (Fig. 3d).

### *ESR1* mutation and *CCND1* amplification were frequently detected in the HR + PDX model.

**Fig. 4:**
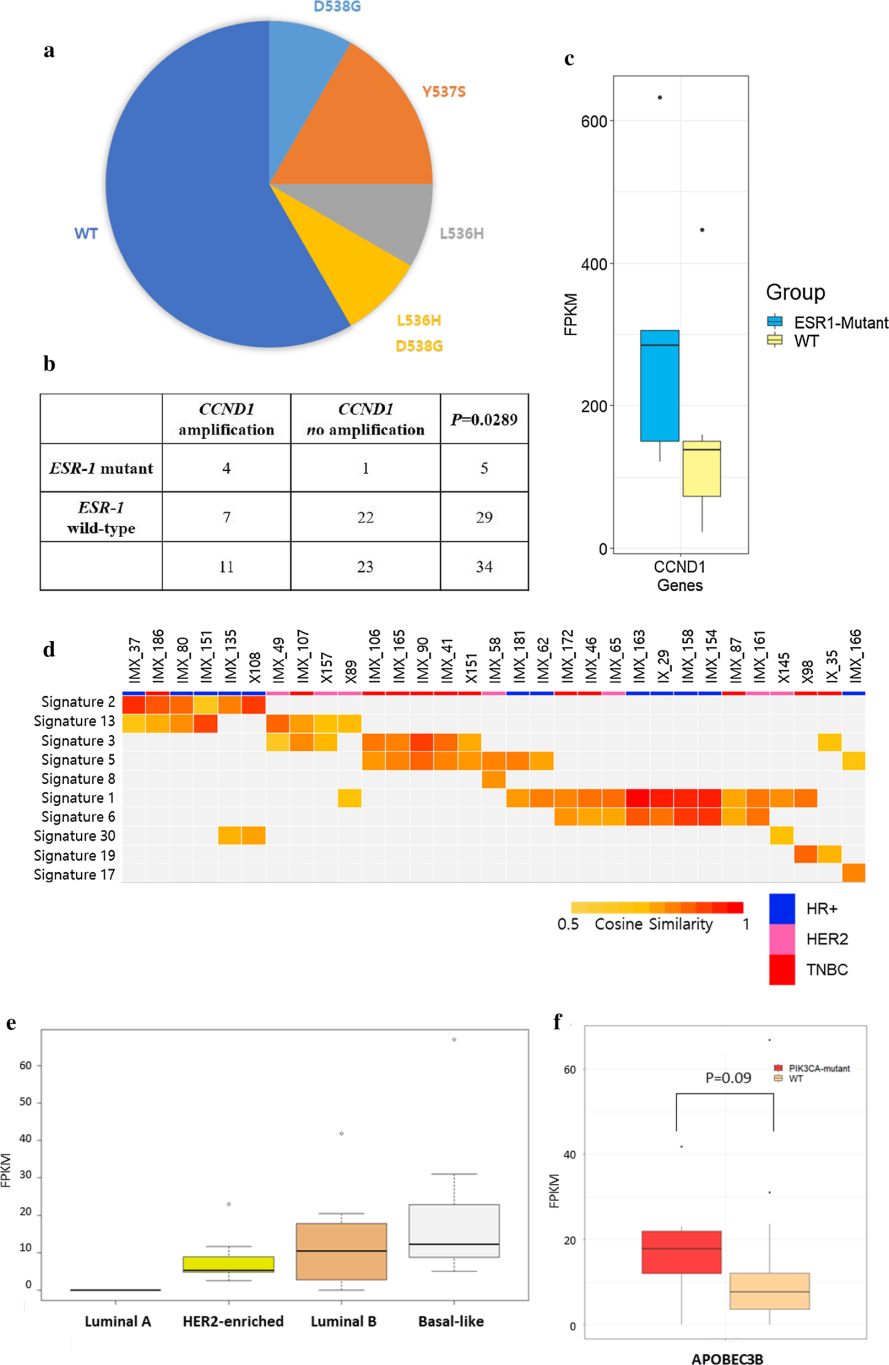
Significant features in the HR + PDX model. **a** *ESR1* mutations in the HR + PDX model. **b** *CCND1* amplification was analyzed by Fisher’s exact test in all of our PDX models. **c** The FPKM values of the *CCND1* gene were analyzed, and those from *ESR1*-mutated samples and wild-type samples were compared. **d** Mutation signature analysis was performed, and the signatures were clustered. The signatures depict the 1st and 2nd highest cosine similarity. **e, f** *APOBEC3B* expression is presented as its FPKM value by subtype (**e**) or the *PIK3CA* gene mutation (**f**)

Establishment of the HR + HER2- breast cancer PDX model was challenging, and the take rate was 2–6.7% [7, 8]. The take rate of the HR + HER2- PDX model was 22% (14/64) in our study. Two cases originated from the same patient; thus, we analyzed 12 cases of the HR + HER2- PDX model. As presented in Fig. 4a, among the 12 PDX cases, 5 (41%) harbored the pathogenic *ESR1* mutation, which was more frequently detected in our HR + HER2- PDX model than in a previous model (Fig. 3b). Interestingly, *CCND1* amplification was associated with the *ESR1* mutation by Fisher’s exact test (Fig. 4b, p = 0.0289). Furthermore, *CCND1* expression was elevated in the *ESR1*-mutated PDX model (Fig. 4c).

### *APOBEC3B* expression was elevated in luminal B subtype and *PIK3CA*-mutant samples

**Fig. 5:**
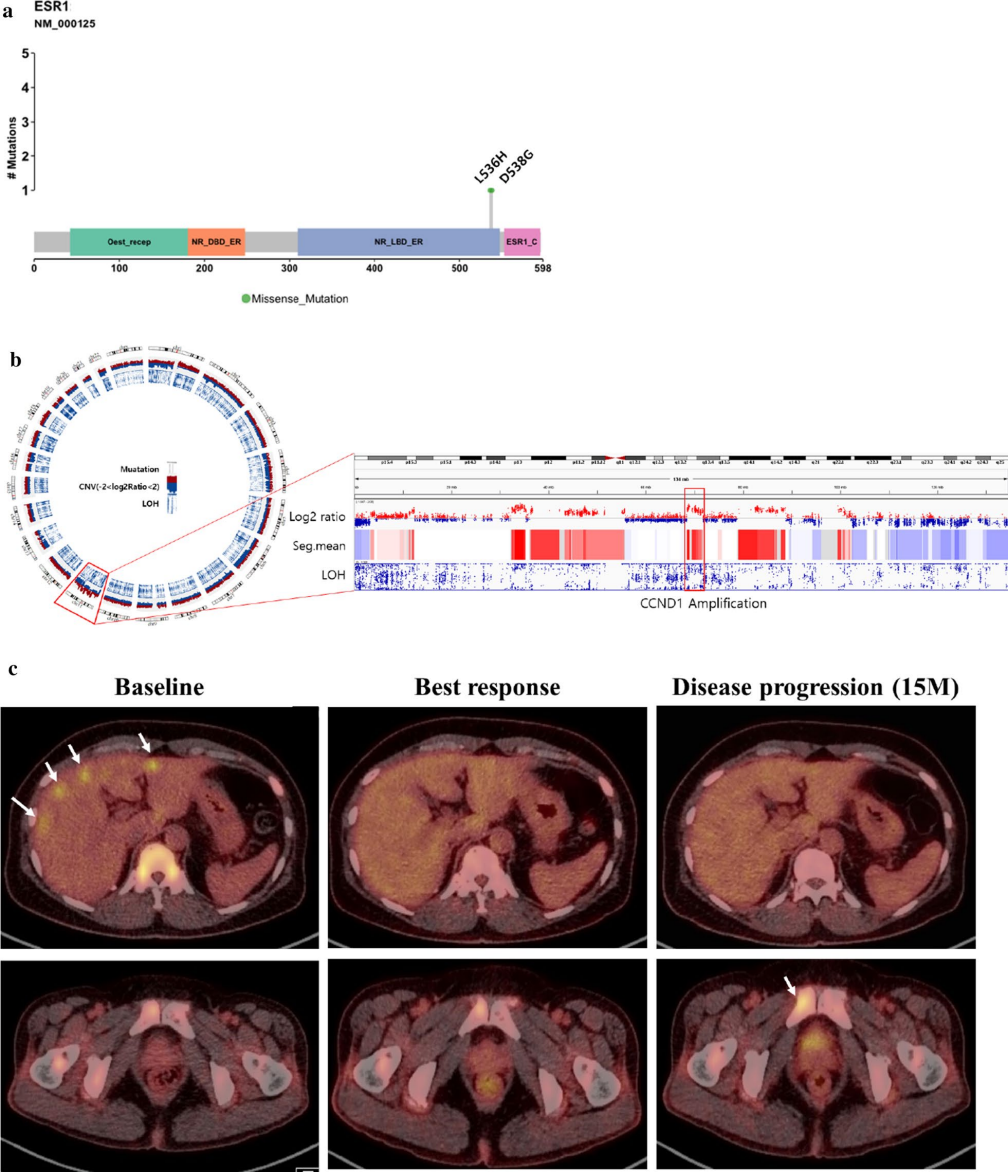

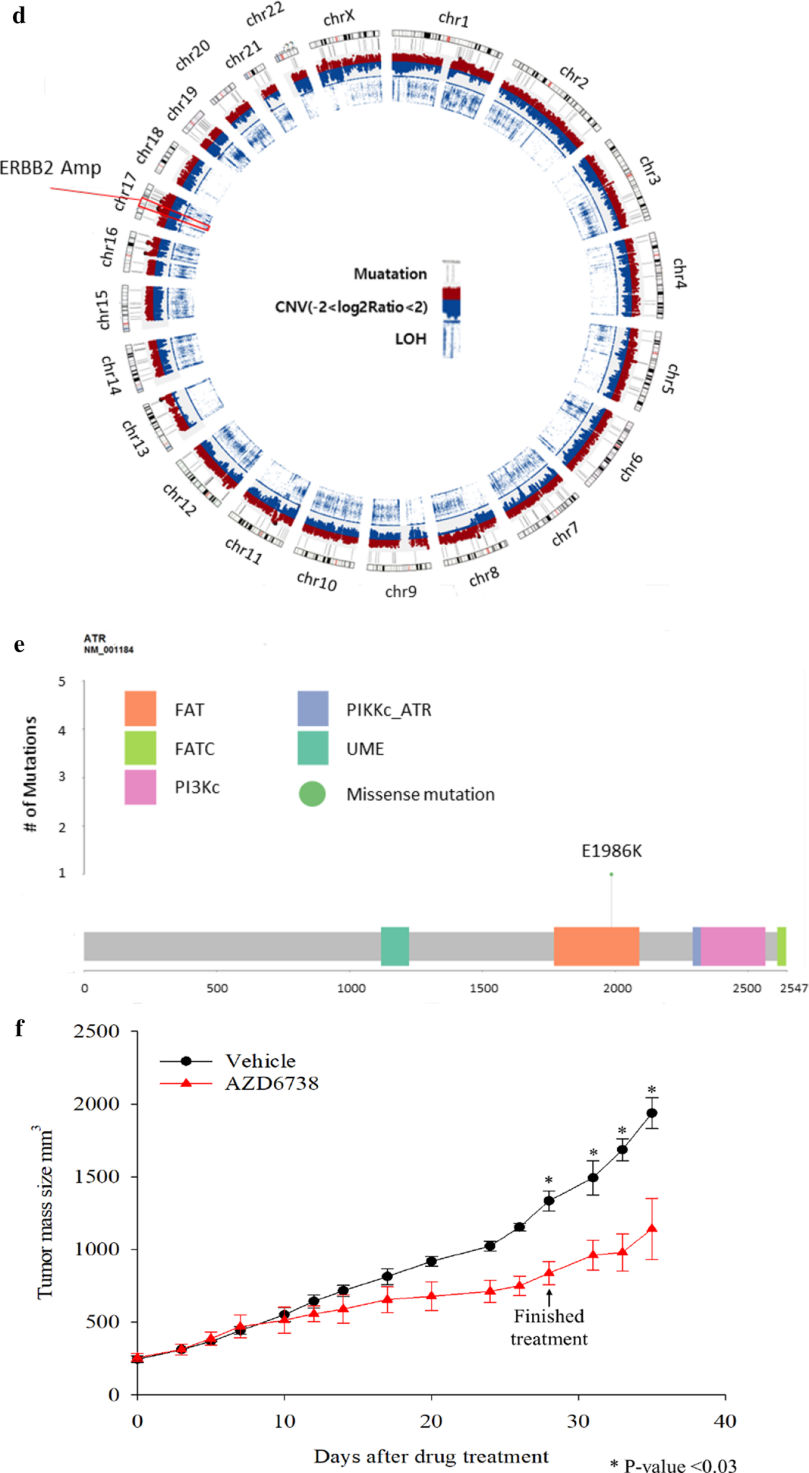
Utility of PDX genomic data and in vivo drug tests for clinical implications. **a** ESR1 mutation site and its domain in the IMX-158 sample. **b** CNV in the IMX-158 sample. **c** PET scan of the donor of the IMX-158 sample. Baseline indicates before the initiation of palbociclib and fulvestrant treatment. The best response was observed four months after initiation. White arrows indicate hypermetabolic lesions in the liver and pelvic bone. **d** CNV in the X89 sample. **e** ATR mutation site and its domain in the X89 sample. **g** The X89 PDX model was treated with the ATR inhibitor AZD6738 for 4 weeks. The tumor volumes are presented as graphs

Recently, mutational signature analysis has been widely used to understand the mutation patterns and underlying biological processes. We performed mutational signature analysis to determine whether our PDX model has a specific mutational signature. The highest and second highest signatures based on cosine similarity were used for clustering. The APOBEC (COSMIC signatures 2 and 13), CpG (COSMIC signature 1), MSI (COSMIC signature 6), and BRCA (COSMIC signatures 3 and 8) signatures were prominent in our models (Fig. 5a). In particular, signature 3 was dominant in the TNBC subtype, whereas the APOBEC signature was prominent in the HR + HER2- subtype, following signature 1 (Fig. 4d).

Since *APOBEC3B* is reported to promote breast cancer cell growth depending on the ER status [27, 28], we analyzed the correlations between the APOBEC signature and success rate of the establishment of the HR+ HER2- PDX model. Among the 12 HR+ HER2- PDXs, 5 (41.7%) had a clustered APOBEC signature. A previous study reported that *APOBEC3B* expression is associated with poor clinical outcomes and proliferative features [29]. Accordingly, we further analyzed *APOBEC3B* expression in our model. Initially, we observed that samples with the APOBEC signature presented high transcription levels of APOBEC family genes. Only the expression level of *APOBEC3B* showed differences among all APOBEC family gene groups, and the APOBEC group showed the highest level (Additional file 1: Fig. S3a). The results of our *APOBEC3B* expression analysis revealed differences between each subtype and *PIK3CA* mutation. We divided the cases into 4 subtypes (luminal A, luminal B, HER2-enriched, and basal like) using transcriptome sequencing data from the PDX samples [20]. *APOBEC3B* expression was the highest in the basal-like subtype, followed by luminal B, HER2, and luminal A (Fig. 4e). This result indicated that our HR + HER2- PDX model exhibited aggressiveness. The relationships between APOBEC activity and *PIK3CA* mutations have been reported in a previous study [30]. In our PDX model, all *PIK3CA*-mutant cases except for one case clustered in the APOBEC signature. Moreover, *APOBEC3B* expression was higher in the *PIK3CA*-mutated samples than in the wild-type samples (Fig. 4f). Therefore, our HR + HER2- PDX model showed an APOBEC-related signature and elevated expression of *APOBEC3B*. The *PIK3CA* mutation seems to correlate with signatures 2 and 13 and the expression of *APOBEC3B*.

### Clinical implications of the PDX model

After performing genomic profiling of our established PDX model, we examined the clinical application of the PDX model and analyzed its genomics.

To demonstrate that PDX genomic profiling can predict the clinical outcome of a disease, we analyzed the genetic features of IMX-158 cases originating from a metastatic liver specimen of a patient who received various endocrine therapies, including tamoxifen, letrozole and exemestane with everolimus. The IMX-158 PDX model harbored two *ESR1* mutation sites in L536H and D538G (Fig. 5a), with *CCND1* amplification (Fig. 5b). The donor of the IMX-158 model was treated with fulvestrant and the CDK4/6 inhibitor palbociclib. After 4 months of treatment, the patient achieved partial response by RECIST 1.1 (Fig. 5c). The maximal standardized uptake value (SUV) with FDG PET/CT was reduced from 8.6 to 6.0 in the liver and 6.8 to 2.5 in the pelvic bone during the treatment. At 15 months, the disease progressed in previously noted bone lesions (maximal SUV from 3.6 to 5.1). Our patient had a longer PFS duration than those in the fulvestrant plus palbociclib group in the phase III PALOMA-3 trial, with a median PFS duration of 9.5 months (95% CI 9.2–11.0 months) [31, 32], and these data suggest that *CCND1* amplification and the *ESR1* mutation might affect the long-term benefits of treatment. Moreover, the genomic analysis of the PDX model may facilitate interpretation of the clinical outcome of a certain treatment.

The X89 model was established from ER + HER2 + subtype mBC. The donor of X89 was treated with several anti-HER2 therapies, including trastuzumab, lapatinib, and margetuximab (MGAH22). The WES data of the PDX model revealed *ERBB2* amplification, similar to the patient data (Fig. 5d). Moreover, this model conferred the somatic mutations *ATR* E1986K and *BRCA2* E1734K (Fig. 5e, Additional file 1: Table S3). Biological function was not reported initially in these two mutations; however, they were revealed to play a pivotal role in the DNA repair pathway. Thus, we adjusted for the ATR inhibitor in this model to evaluate drug efficacy. PDX tumor growth was delayed in the ATR treatment group compared with the vehicle group (Fig. 5f). These results indicate that the administration of an ATR inhibitor to ATR-mutated tumors could be a good treatment option. Additionally, PDX models could be a useful tool for testing drug sensitivity in vivo and provide strong evidence to expand the drug’s clinical indications.

## Discussion

Breast cancer is highly heterogeneous, not only across the patient population but also in intratumoral features. The NGS technique has been used to understand the behavior of breast cancer, and the genomic alterations involved in tumorigenesis or tumor progression have been extensively described; however, these efforts focused only on early-stage breast cancer [33, 34]. Although several mutation profiles of mBC are available [26], they are insufficient to completely understand breast cancer characteristics. Moreover, numerous novel agents have shown better responses than those used previously, but the response rate to systemic chemotherapy is approximately 50% to 90% for primary tumors [35]. Thus, it is essential to reveal the genomic profile of metastatic tissue and to explore an efficient therapeutic strategy for patients with mBC.

The PDX model is known to play a pivotal role in retaining the molecular and biological features of donated tumor tissue [8]. The CNV and exome sequencing data demonstrate high fidelity between the paired samples [6]. Moreover, drug efficacy tests using the PDX model have shown promising results; a test of cetuximab and gemcitabine using the PDX model yielded results similar to those obtained in clinical trials [36–38]. Nevertheless, the PDX model has certain limitations. As not all PDX implantations lead to PDX model establishment, an improvement in the PDX take rate is a major issue. In particular, because of the low take rate of the HR + HER2- PDX model, reports analyzing genomic and ethnic diversity in the HR + HER2- model are not sufficient. In this study, we report a take rate of 26% using core needle biopsy specimens (Fig. 2a). In contrast to previous studies [6, 7, 39, 40], the take rate of the HR + HER2- PDX model in our system was significantly high (22%). The patients enrolled in this study had mBC, which is considered an aggressive form of breast cancer. Among the established PDX models, 67.6% of donor tumor tissues were histology grade 3. In addition, we aimed to create an efficient and rapid system to establish PDX models. Most (86%) fresh tumor biopsy tissues were delivered to the implant team within 30 min and implanted within an hour. We speculate that these factors might have affected the elevated take rate in our system.

Model fidelity was analyzed between the paired tumor tissues from donors and those from the PDX model. The tumor subtype based on the IHC results showed 90% concordance (Fig. 2d). The somatic mutation and CNV fidelity between the donor and PDX models were also well matched (Fig. 2e, f). The take rate of the proliferative tumor was higher in our PDX model (Fig. 2c). Additionally, we further analyzed the Ki-67 index in the HR + HER2- PDX model, and the Ki-67 index was higher in graduation cases than in fail cases (Additional file 1: Fig. S1b). The PDX model presented a higher frequency of *TP53* and *ESR1* mutations, as well as a higher tumor mutation burden than other metastatic regimens. The somatic mutation allele frequency tended to be higher in the PDX model. This might have been due to clonal composition changes or as a result of increased human tumor DNA purity in the PDX model [6].

We also analyzed the RNA expression pattern using PDX tumor tissue. Unsupervised hierarchical clustering analysis revealed that each sample was clustered by its respective subtype (Additional file 1: Fig. S4a). HR + HER2- and HER2 + samples tended to cluster into one group, whereas the TNBC samples clustered into another. We further analyzed ontologies based on the differentially expressed gene (DEG) analysis (Additional file 1: Fig. S4b). g:Profiler was used to analyze and deduct ontologies using REVIGO. Enriched pathways were associated with blood vessel development, cell proliferation, cell adhesion, and extracellular matrix organization. In contrast, the pathways related to cell projection organization and hormone transport were downregulated (Additional file 1: Fig. S4c). In general, the RNA expression profile tended to cluster based on the subtype of each sample, and the proliferation and cell adhesion-related ontologies were upregulated in the TNBC-dominant cluster.

Our HR + HER2- PDX model is better established than the previously reported PDX model using therapy-naïve biopsy samples (22% vs 6.7%, [7]). Among the 12 hormone-positive PDX models, 5 harbored *ESR1* mutations (41%). Moreover, all mutations that we found are known pathogenic mutations (L536H, Y537S, and D538G). The donors of our PDX model were usually heavily pretreated using many lines of therapy, including aromatase inhibitors. The mutational burden of our PDX model tended to be higher than that of the database. This also might have contributed to the high take rate in this study. As the amount of the needle biopsy specimen was limited, we did not have available remaining tissue for the failed cases.

The APOBEC signature and the high expression of *APOBEC3B* were prevalent in our PDX model. The HR + HER2- PDX model clustered into aging- and APOBEC-related signatures, and the mutation patterns were also matched. Moreover, *APOBEC3B* expression was upregulated in the luminal subtype and *PIK3CA*-mutated samples. The *PIK3CA* mutation samples were clustered into signatures 2 and 13. The relationship between the APOBEC signature and the *PIK3CA* mutation is well reported, but that between *APOBEC* gene expression and the mutational signature remains unclear. From the recent report of Cescon DW et al., *APOBEC3B* expression is related to a lack of ER expression and to the expression of key proliferation-associated genes (*AURKA*, *MK167* and *CCNB1*) [27, 29]. Based on these data, we surmised that the metastatic tissue of HR + HER2- tumors tends to have a more aggressive phenotype; thus, the take rate of our HR + HER2- PDX was higher than that described in previous reports.

The PDX model can be efficiently used for translational research. IMX-158, an ER + HER2- breast cancer model, harbors an *ESR1* mutation and *CCND1* amplification. The *ESR1* D538G mutation was reported to promote the estrogen-independent activation of estrogen receptor [41]. Moreover, mBCs with *ESR1* mutations are associated with a poor prognosis and are not highly responsive to aromatase inhibitors [42]. However, this mutation was shown to elicit a response to fulvestrant treatment in mouse models and in clinical trials [43]. In addition to the *ESR1* mutation, *CCND1* amplification might also affect treatment decisions. Cyclin D1 is a binding partner of CDK4/6, the target of palbociclib, and regulates the cell cycle in G1 phase [44]. Cyclin D1 is a transcriptional target of ER [45], and *CCND1* amplification is found in 15% of breast cancers [46]. Moreover, over 50% of breast cancer cells overexpress cyclin D1 [46]. The response to palbociclib in cell lines is associated with cyclin D1 expression [47]. Based on these studies, the donor of this model was treated with fulvestrant and palbociclib. This patient experienced a prolonged progression-free survival (PFS) duration of 15 months, which was longer than the median PFS duration reported in the PALOMA-3 trial (9.5 months; 95% CI 9.2–11.0 months). This case highlights that genomic analysis of the PDX model can be utilized for treatment selection and the prediction of clinical outcomes. Moreover, the PDX genomic profile and the PDX model itself can contribute to translational research. In drug tests using X89, an HR- HER2 + PDX model, genomic analyses of the PDX model could be used to expand a drug's indication to unknown somatic mutations. These results also implicate that our PDX models could be helpful to test the sensitivity of specific drugs in vivo and provide some evidence to facilitate their clinical application.

## Conclusion

A hormone-positive breast cancer PDX model was established in this study. The genomic profile of PDX tissue well reflects the characteristic of donor tissue. The *ESR1* mutation, *CCND1* amplification, and the APOBEC signature, which represent the aggressive phenotype, are outstanding in our HR + HER2- PDX model. Moreover, our PDX model proved its potential for use in clinical implications.

## Supplementary information

**Additional file 1: Figure S1**. a. Immunohistochemical staining was conducted using PDX tumors. The tissue subtype of each patient is shown at the top of the figure. b. The box plot shows a comparison of the Ki-67 indexes of established and failed cases of the HR+ HER2- PDX model. c. The CNVs in individual samples are shown as a bar chart. The case number with IMT indicates the results of patient tissue analysis, and the case number with IMX indicates the results of PDX tissue analysis.** Figure S2**. a. The frequency of somatic mutations was compared with the other datasets, and the data were aligned in order from the most frequent dataset. The data obtained from "the metastatic breast cancer project" represented 2017 MBC, and the dataset of “the SAFIR01, SAFIR02, SHIVA, or Molecular Screening for Cancer Treatment Optimization (MOSCATO) prospective trials" represented 2015 IGR.** Figure S3.** a. The expression of the APOBEC family gene is illustrated with its FPKM values by signature group.** Figure S4**. a. Unsupervised hierarchical clustering results of our PDX models are presented. The samples were clustered by two groups: ‘Group A’ and 'Group B'. Group A is a cluster of mainly HR+ subtype and HER2+ subtype samples. Group B is a cluster of mainly TNBC subtype samples. b. The DEG analysis results are shown in this figure. The upregulated genes of the B group are depicted as red dots, and the downregulated genes of the B group are depicted as blue dots. c. Gene ontology based on the DEG analysis was performed with g:Profiler and clustered with REVIGO. The log10 p-value is illustrated by color.** Table S1.** The metastatic tumor tissue biopsy site.** Table S2. **The origin of the biopsy tissues and pathologic characteristics of established PDX specimens are described according to the tumor subtype.** Table S3**. Mutation information of X89.

**Additional file 2.** The information on sequencing data.

## Data Availability

The datasets used and/or analyzed during the current study are available from the corresponding author on reasonable request.

## Abbreviations

PDX: Patient-derived xenograft
mBC: Metastatic breast cancer
TNBC: Triple-negative breast cancer
ER: Estrogen receptor
PR: Progesterone receptor
HER2: Human epidermal growth factor receptor 2
WES: Whole exome sequencing
CNV: Copy number variation
HR: Hormone receptor
IDC: Infiltrating ductal carcinoma
ILC: Invasive lobular carcinoma
IHC: Immunohistochemistry
PFS: Progression-free survival
