# Supplementary material for: Genomic profile of metastatic breast cancer patient-derived xenografts established using percutaneous biopsy
Source: J Transl Med. 2021 Jan 6;19:7. doi: 10.1186/s12967-020-02607-2 (PMC7789010; doi:10.1186/s12967-020-02607-2)
Supplement: Supplementary file 1 — Additional file 1: Figure S1. a. Immunohistochemical staining was conducted using PDX tumors. The tissue subtype of each patient is shown at the top of the figure. b. The box plot shows a comparison of the Ki-67 indexes of established and failed cases of the HR+ HER2- PDX model. c. The CNVs in individual samples are shown as a bar chart. The case number with IMT indicates the results of patient tissue analysis, and the case number with IMX indicates the results of PDX tissue analysis. Figure S2. a. The frequency of somatic mutations was compared with the other datasets, and the data were aligned in order from the most frequent dataset. The data obtained from "the metastatic breast cancer project" represented 2017 MBC, and the dataset of “the SAFIR01, SAFIR02, SHIVA, or Molecular Screening for Cancer Treatment Optimization (MOSCATO) prospective trials" represented 2015 IGR. Figure S3. a. The expression of the APOBEC family gene is illustrated with its FPKM values by signature group. Figure S4. a. Unsupervised hierarchical clustering results of our PDX models are presented. The samples were clustered by two groups: ‘Group A’ and 'Group B'. Group A is a cluster of mainly HR+ subtype and HER2+ subtype samples. Group B is a cluster of mainly TNBC subtype samples. b. The DEG analysis results are shown in this figure. The upregulated genes of the B group are depicted as red dots, and the downregulated genes of the B group are depicted as blue dots. c. Gene ontology based on the DEG analysis was performed with g:Profiler and clustered with REVIGO. The log10 p-value is illustrated by color. Table S1. The metastatic tumor tissue biopsy site. Table S2. The origin of the biopsy tissues and pathologic characteristics of established PDX specimens are described according to the tumor subtype. Table S3. Mutation information of X89. [file 12967_2020_2607_MOESM1_ESM.pptx]

## Slide 1
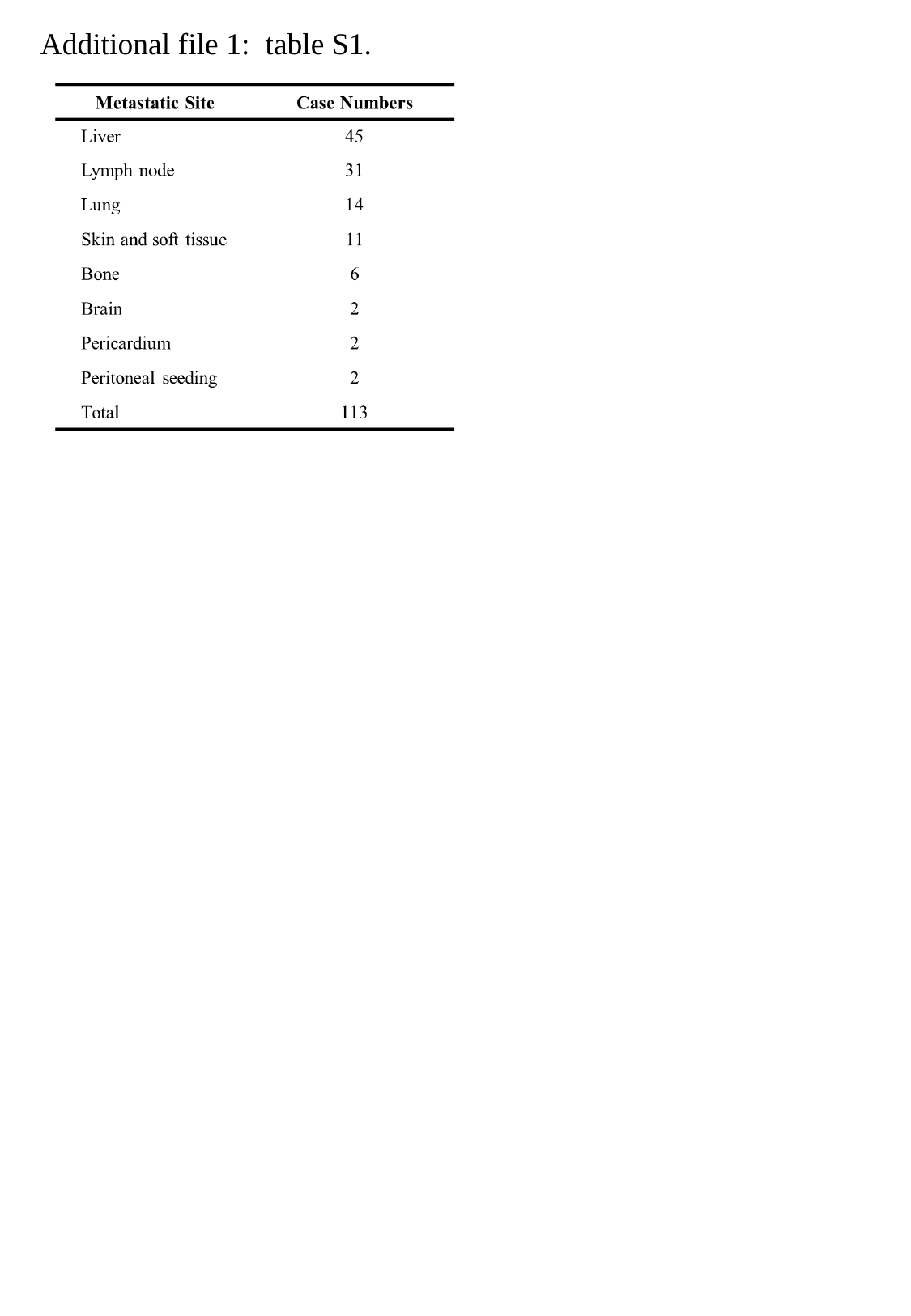

Additional file 1: table S1.

## Slide 2
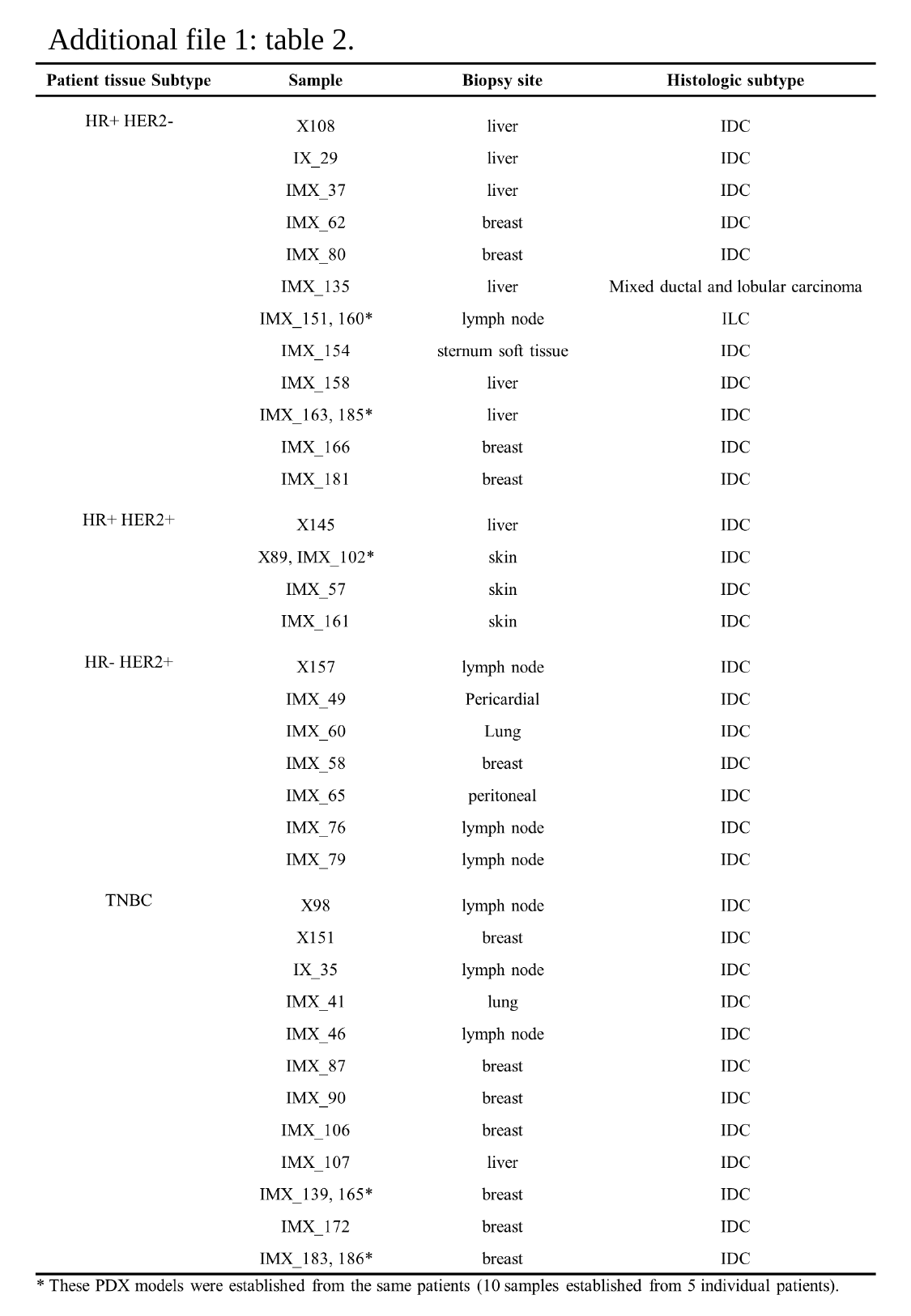

Additional file 1: table 2.

## Slide 3
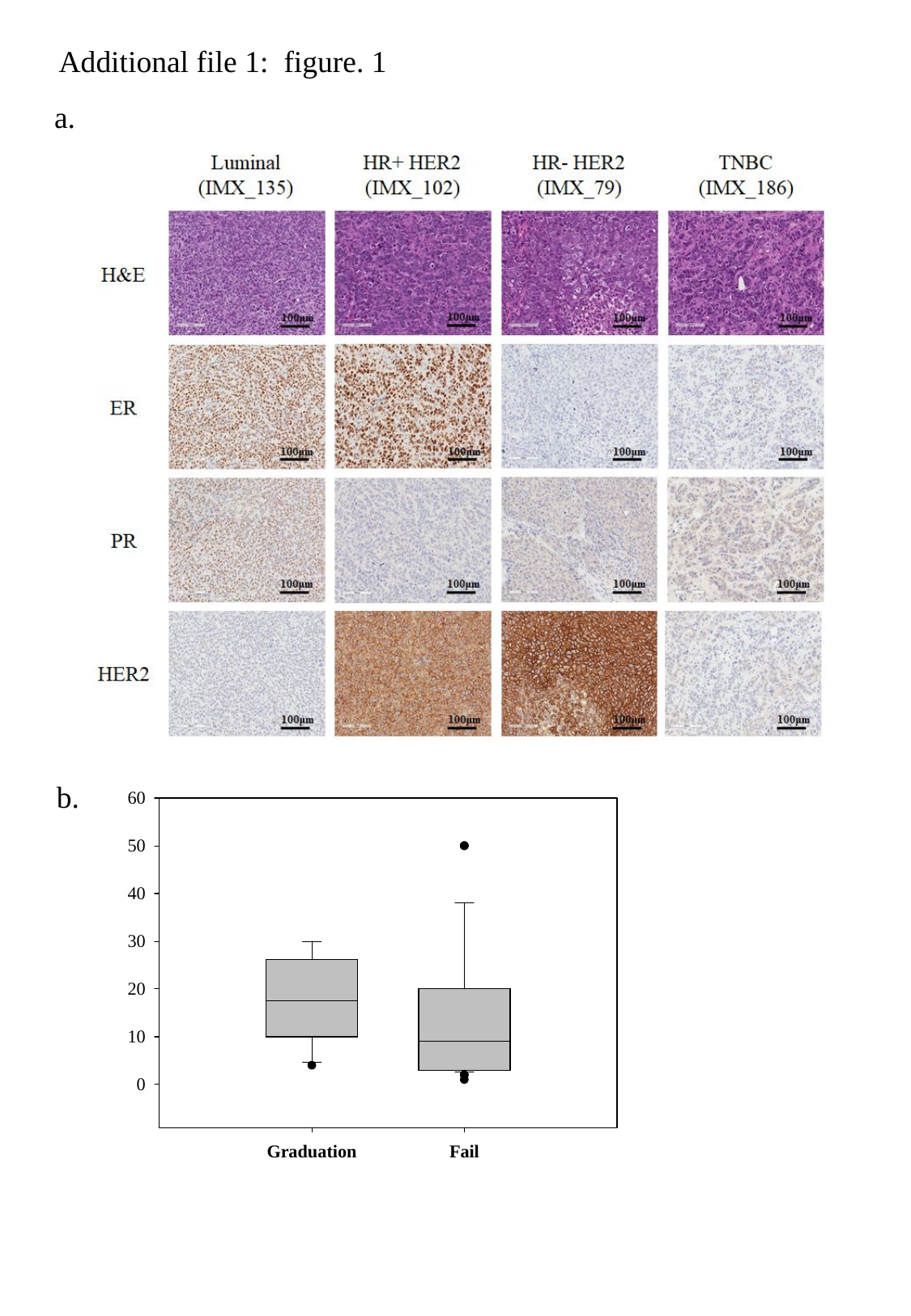

Additional file 1: figure. 1
a.
b.

## Slide 4
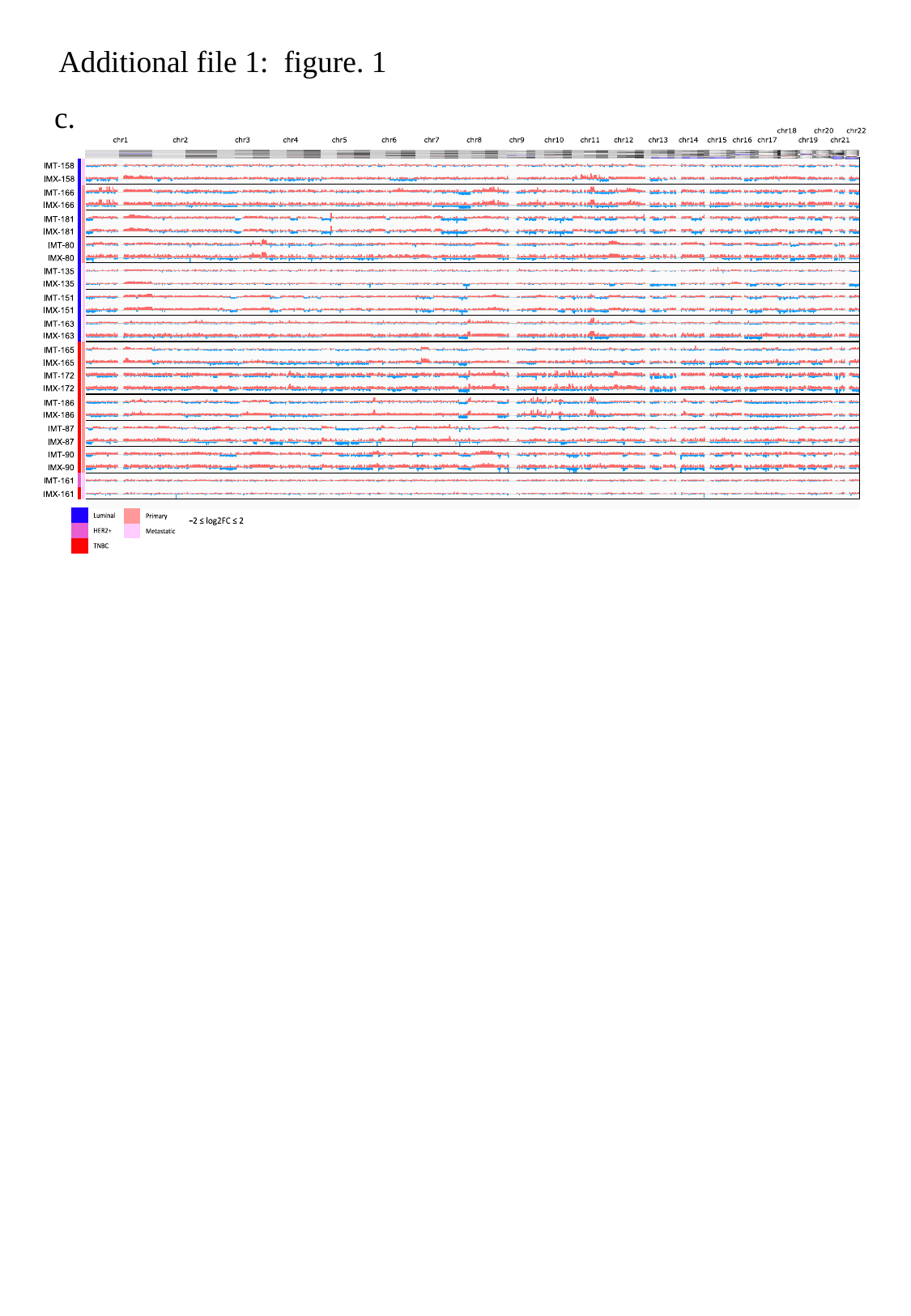

Additional file 1: figure. 1
c.

## Slide 5
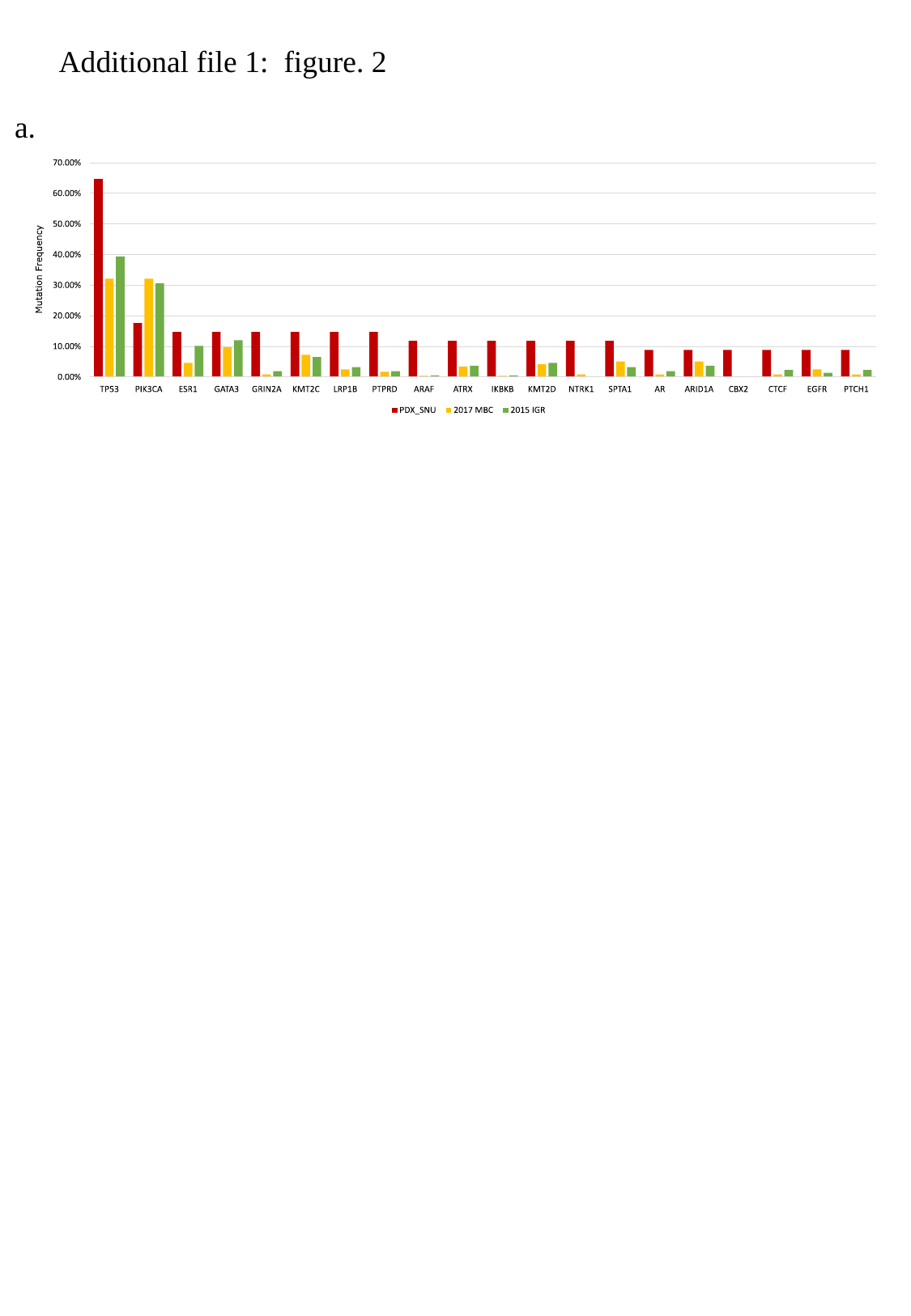

Additional file 1: figure. 2
a.

## Slide 6
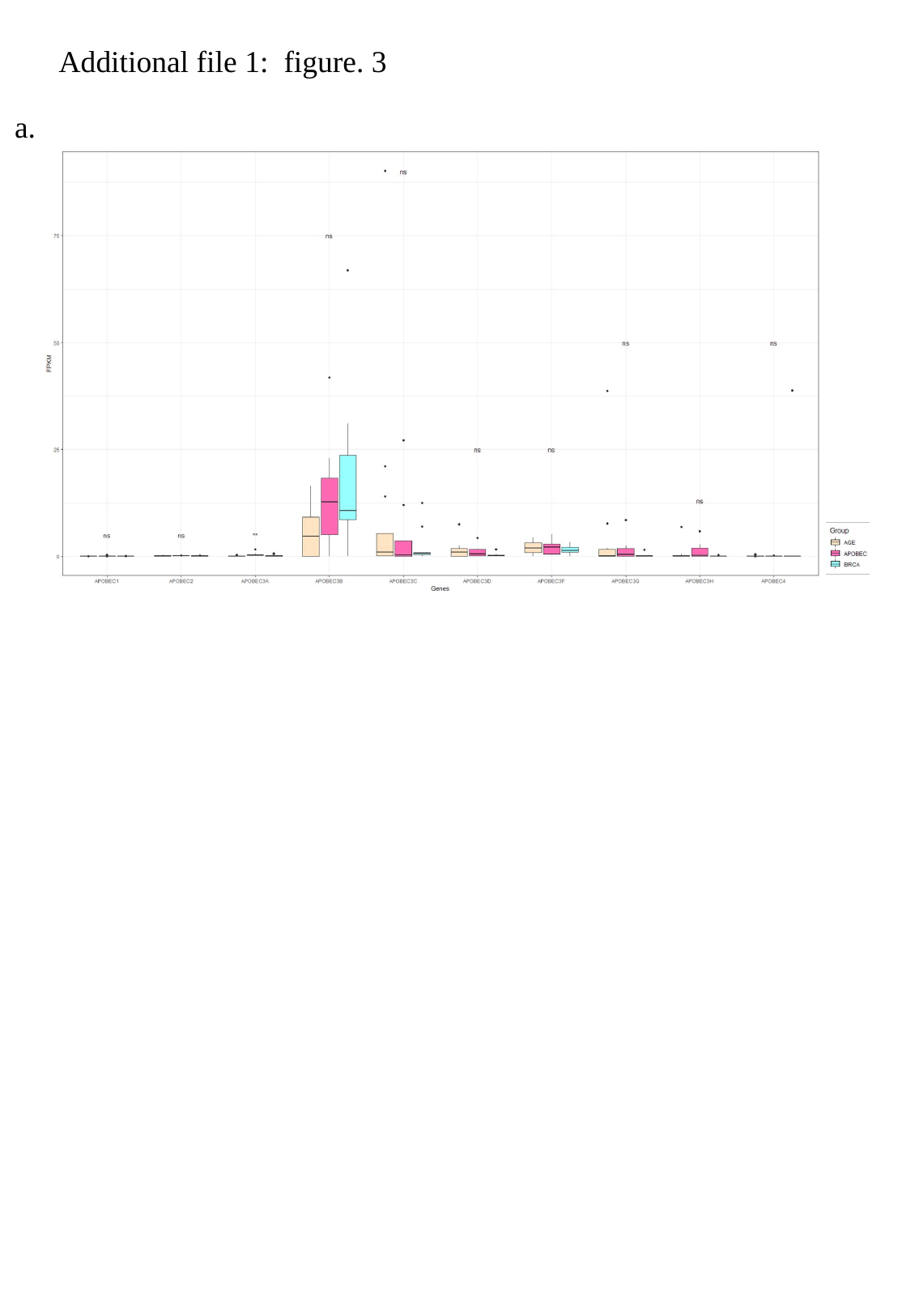

Additional file 1: figure. 3
a.

## Slide 7
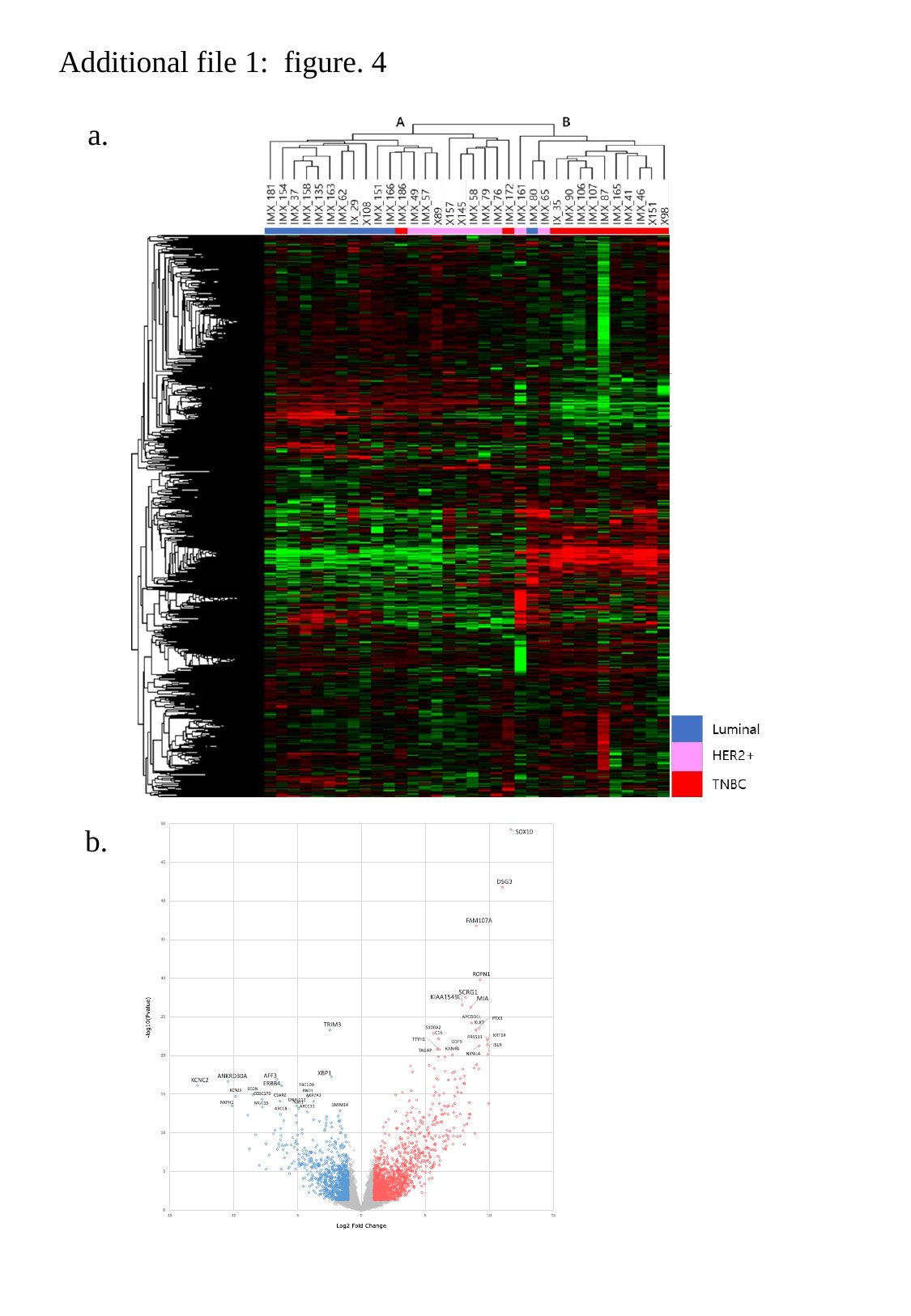

Additional file 1: figure. 4
a.
b.

## Slide 8
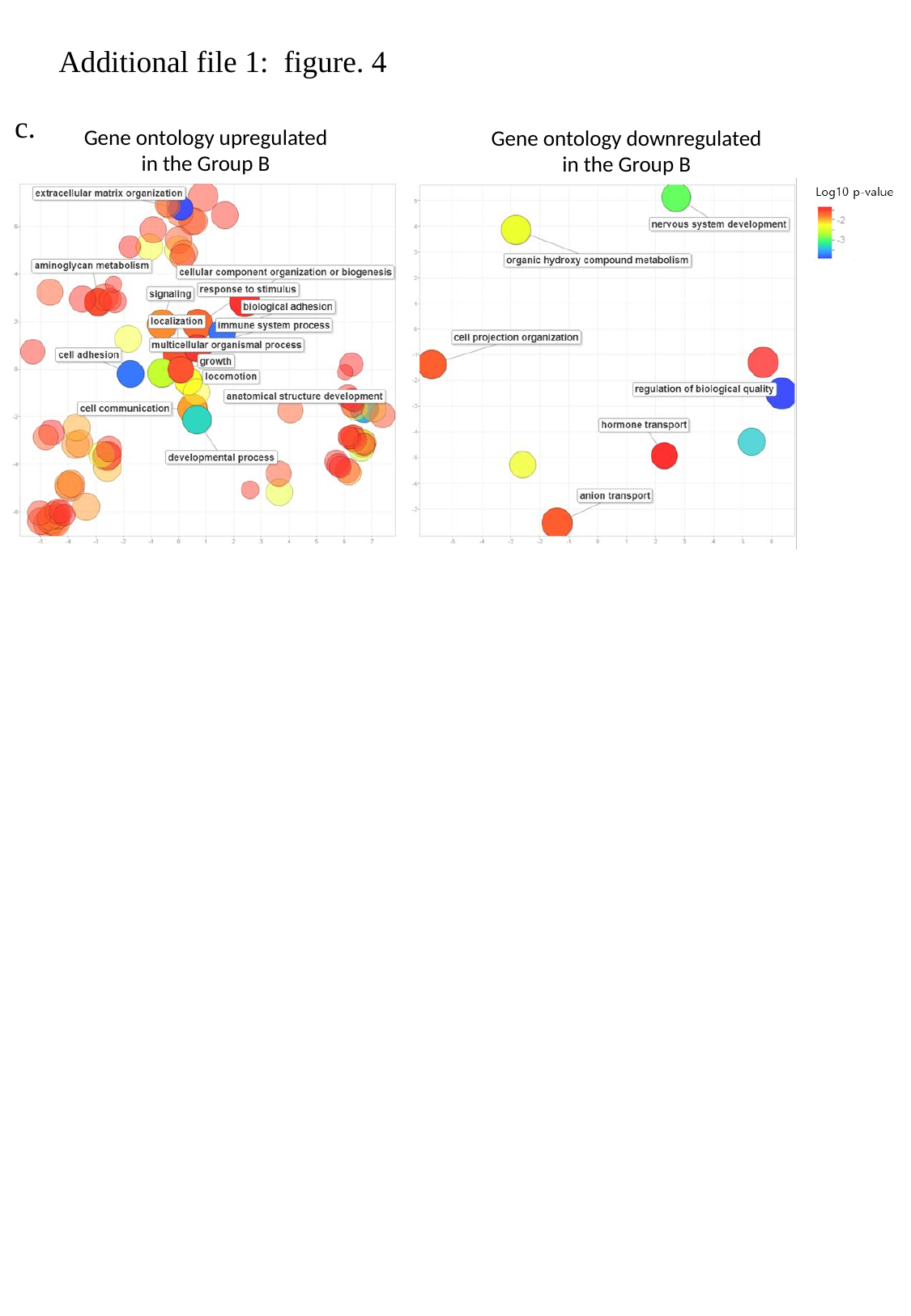

Additional file 1: figure. 4
c.
Gene ontology upregulated in the Group B
Gene ontology downregulated in the Group B

## Slide 9
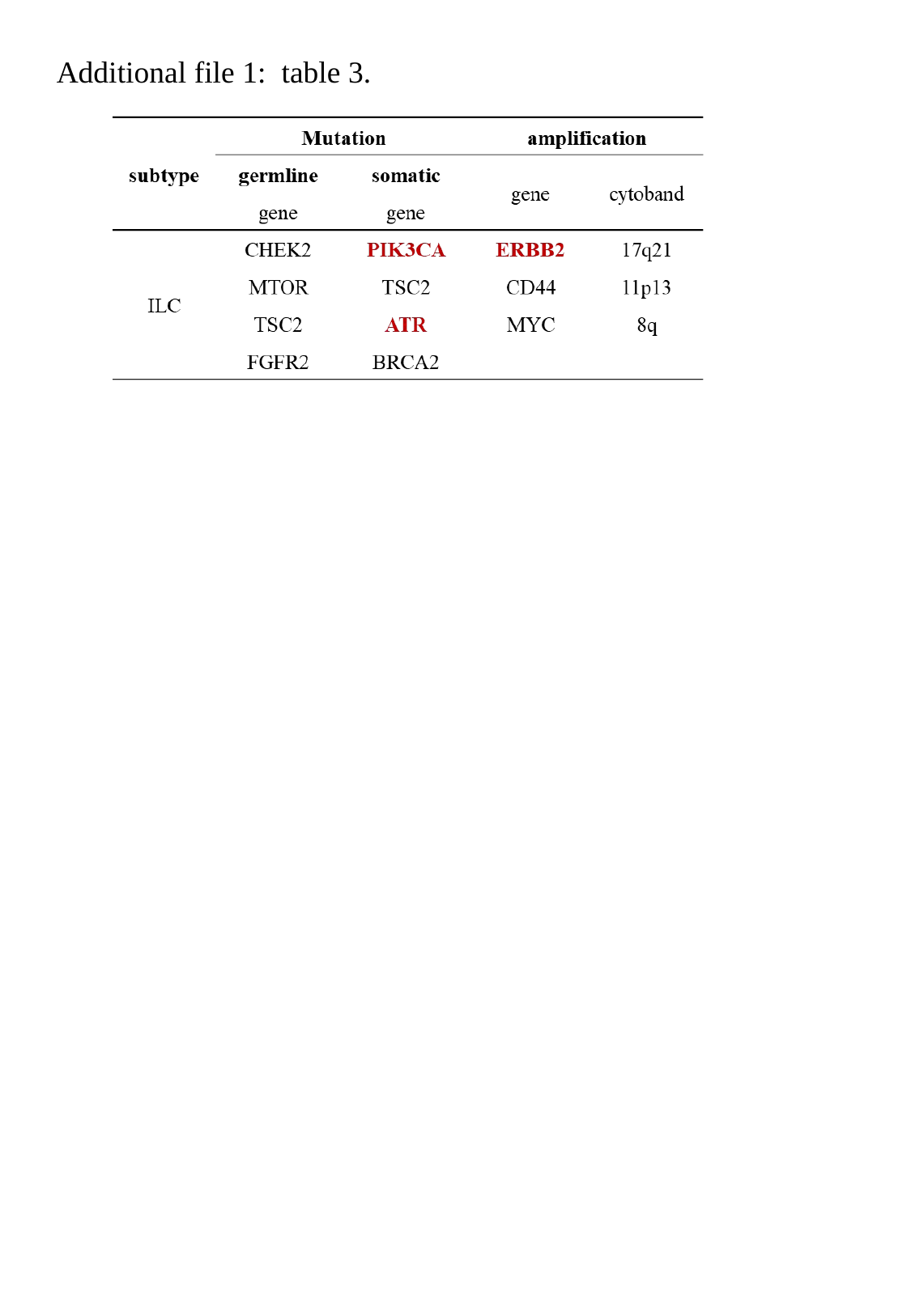

Additional file 1: table 3.
